# Femoral fracture in the elderly: dependence on nursing care

**DOI:** 10.15649/cuidarte.3186

**Published:** 2024-05-23

**Authors:** Augusto Baisch de Souza, Daniela Tonroller de Oliveira, Sidiclei Machado Carvalho, Jonas Michel Wolf, Tiago Claro Maurer, Lucas Henrique Rosso

**Affiliations:** 1 Faculdade Ciências da Saúde Moinhos de Vento, Porto Alegre, Brasil. augustobaisch@gmail.com Faculdade Ciências da Saúde Moinhos de Vento Porto Alegre Brasil augustobaisch@gmail.com; 2 Hospital Moinhos de Vento, Porto Alegre, Brasil. daniela.oliveira@hmv.org.br Hospital Moinhos de Vento Hospital Moinhos de Vento Porto Alegre Brazil daniela.oliveira@hmv.org.br; 3 Hospital Moinhos de Vento, Porto Alegre, Brasil. sidiclei.carvalho@hmv.org.br Hospital Moinhos de Vento Hospital Moinhos de Vento Porto Alegre Brazil sidiclei.carvalho@hmv.org.br; 4 Hospital Moinhos de Vento. jonas.wolf@hmv.org.br Hospital Moinhos de Vento Hospital Moinhos de Vento Brazil jonas.wolf@hmv.org.br; 5 Hospital Moinhos de Vento. tiago.maurer@hmv.org.br Hospital Moinhos de Vento Hospital Moinhos de Vento Brazil tiago.maurer@hmv.org.br; 6 Faculdade Ciências da Saúde Moinhos de Vento, Porto Alegre, Brasil. lucas.rosso@hmv.org.br Faculdade Ciências da Saúde Moinhos de Vento Porto Alegre Brasil lucas.rosso@hmv.org.br

**Keywords:** Nursing Assessment, Classification, Nurses Improving Care for Health System Elders, Femoral Fracture, Health of the Elderly, Evaluación en Enfermería, Clasificación, Nurses, Enfermeras que Mejoran la Atención de los Ancianos en el Sistema de Salud, Fracturas del Fémur, Salud del Anciano, Avaliação em Enfermagem, Classificação, Cuidado de Enfermagem ao Idoso Hospitalizado, Fraturas do Fêmur, Saúde do Idoso

## Abstract

**Introduction:**

Due to the aging of the population, nursing processes have been adapted to these patients, who require a high level of care and guidance.

**Objective:**

Analyzing the degree of dependence on nursing care by elderly patients (65 years or older) with femur fractures.

**Materials and Methods:**

retrospective, with a quantitative approach, carried out in a private hospital from April 2021 to April 2022. According to the Patient Classification System (PCS), the sample comprehends 41 patients, analyzed epidemiological data and degree of dependence Study of nursing care during hospitalization, environment of hospitalization, and discharge.

**Results:**

Composed of 41 patients, mean age of 84 years and female predominance (75.61%). Concerning fractures, there was a greater occurrence due to falls from standing height and a predominance of neck fractures, with an average time until surgery of less than 16 hours. Systemic Arterial Hypertension and Diabetes Mellitus were predominant. The average of the PCS estimates presented 24.26 in the 1st, 26.12 in the 2nd, and 26.24 in the 3rd. The length of hospital stay was 7 days and no deaths were reported.

**Discussion:**

The findings on sociodemographic data, reasons for falls, location, comorbidities, degree of dependence, and length of hospital stay are similar to those available in databases. They differ, in better quality, under time until surgery and clinical.

**Conclusions:**

The study presents specific knowledge to carry out the care of the intra hospital nursing process, thus allowing the systematization of the team’s assistance.

## Resumen

**Introducción:**

Debido al envejecimiento de la población, los procesos de enfermería se están adaptando a estos pacientes, quienes requieren un alto grado de cuidado y orientación.

**Objetivo:**

Analizar el grado de dependencia de los cuidados de enfermería por parte de pacientes ancianos (65 años o más) con fracturas de fémur.

**Materiales y Métodos:**

Estudio retrospectivo, con enfoque cuantitativo, realizado en un hospital privado desde abril de 2021 a abril de 2022. La muestra está compuesta por 41 pacientes, se analizaron datos epidemiológicos y grado de dependencia de los cuidados de enfermería al ingreso, media hospitalización y alta, según el Sistema de Classificacao de Pacientes (SCP)

**Resultados:**

Compuesto por 41 pacientes, edad promedio de 84 años y predominio del sexo femenino (75,61%). En cuanto a las fracturas, hubo mayor ocurrencia de caídas desde la altura y predominio de las fracturas de cuello, con un tiempo promedio hasta la cirugía menor a 16 horas. Predominaron la Hipertensión Arterial Sistémica y la Diabetes Mellitus. El promedio de las valoraciones del SCP fue de 24,26 en la 1^a^, 26,12 en la 2^a^ y 26,24 en la 3^a^. El tiempo en hospitalización fue de 7 días y no hubo fallecimientos.

**Discusión:**

Los hallazgos sobre datos sociodemográficos, motivos de caídas, localización, comorbilidades, grado de dependencia y duración de hospitalización son similares a los disponibles en las bases de datos. Se diferencian en términos de mejor calidad, tiempo hasta la cirugía y resultado clínico.

**Conclusión:**

El estudio presenta conocimientos específicos para la realización del cuidado en el proceso de enfermería hospitalaria, permitiendo así sistematizar la asistencia en equipo.

## Resumo

**Introdução:**

Em virtude do envelhecimento da população, os processos de enfermagem estão se adaptando a esses pacientes, os quais necessitam de elevado grau de cuidado e orientação.

**Objetivo:**

Analisar o grau de dependência aos cuidados de enfermagem por pacientes idosos (65 anos ou mais) com fratura de fêmur.

**Materiais e Métodos:**

Estudo retrospectivo, com abordagem quantitativa, realizado em um hospital privado no período de abril de 2021 a abril de 2022. A amostra compreende 41 pacientes, analisados dados epidemiológicos e grau de dependência dos cuidados de enfermagem na admissão, meio da internação e alta, conforme o Sistema de Clasificación de Pacientes (SCP).

**Resultados:**

Composto por 41 pacientes, idade média de 84 anos e predomínio do sexo feminino (75,61%). Sobre a fratura, verificou-se maior ocorrência por queda da própria altura e predominância de fratura de colo, com tempo médio até cirurgia menor que 16 horas. Hipertensão Arterial Sistêmica e Diabetes Mellitus foram predominantes. A média das avaliações do SCP apresentou 24,26 na 1a, 26,12 na 2a e 26,24 na 3a. O tempo de permanência hospitalar foi de 7 dias e não houve nenhum óbito.

**Discussão:**

Os achados sobre os dados sociodemográficos, motivos das quedas, localização, comorbidades, grau de dependência e tempo de permanência hospitalar assemelham-se com os disponibilizados em bancos de dados. Diferem-se, em melhor qualidade, sob tempo até cirurgia e desfecho clínico.

**Conclusões:**

Oestudo apresenta conhecimentos específicos pararealizar ocuidado do processo de enfermagem intra-hospitalar, permitindo, assim, a sistematização da assistência da equipe.

## Introduction

Population aging is a reality in developed and developing countries, leading to an exponential increase in the elderly population. The latest study carried out by the Continuous National Household Sample Survey, published by the Brazilian Institute of Geography and Statistics (IBGE), in which those aged 60 years or over are considered elderly, revealed that in Brazil, in 2012, there were 26.6 million elderly people. In 2018, this number increased by approximately 24%, resulting in 32.1 million, of the total population of 208.5 million^1^.

On an international scenario, the World Health Organization (WHO) estimate shows that, in the year 2025, there will be approximately 1.2 billion elderly people in the world, and around 2 billion in the period from 2050, 80% of which will be in developing countries^2^.

In this context, the increase in the occurrence of femur fractures stands out, as approximately 28% to 35% of individuals over 65 years of age fall each year. A process that normally occurs as a result of trauma such as low energy, is associated with intrinsic and extrinsic factors. The first is evidenced by cognitive decline, malnutrition, reduced functional mobility, decreased visual acuity, use of medications, decreased balance and muscle strength, health problems, and advanced age itself. Extrinsic factors are related to the environment in which the elderly person finds themselves, such as uneven surfaces, slippery floors, unstable tables and chairs, incorrect footwear, and inadequate lighting^3^^,^^5^.

Femur fractures can occur in three regions. Firstly, the proximal region (or epiphysis), divided into head, neck, trochanters, intertrochanteric crest and intertrochanteric line, followed by the diaphysis region, being the middle portion of the bone, extending from the lesser trochanter to the femoral condyles, and by Finally, the distal region, being formed by the medial and lateral condyles, intercondylar fossa and patellar surface^6^.

With their high level of severity, fractures cause major impacts, on the patient's life, their family members, and/or the surrounding community. After the trauma, the elderly experience a decrease in their quality of life, evidenced by the impairment of daily activities, loss of walking without assistance, decreased physical functionality, implications for mental health and psychological issues, increased institutionalization, and, in worst cases, the injury is potentially fatal, resulting in death^7^. According to the National Institute of Traumatology and Orthopedics, 30% to 40% of elderly people will no longer be able to live independently and 20% die within a year of the injury, mainly due to the worsening of previous problems^8^.

Due to the progressive increase in the elderly population, nursing processes are adapting to these patients, as they require a high degree of care and guidance^9^. During the hospital stay, nurses classified the patient's nursing care daily, using the Patient Classification System (SCP). According to the resolution of the Federal Nursing Council (COFEN) number 543/2017, it is the way to determine the patient's degree of dependence on the nursing team, aiming to establish the time spent in direct and indirect care, as well as the qualitative of personnel to meet the patient's biopsychosocial-spiritual needs^10^.

This classification was developed in the United States, in the 1930s, to analyze the differences between patients and the amount of resources needed for care, based on medical diagnosis, sex, and age, which, over the years, changes were added to focus on assistance, establishing the patient's profile and their need for care demands^10^.

Currently, it is divided into 5 titles, namely: 1 - Minimum care patient (PCM): stable patient from a clinical and nursing point of view and self-sufficient in meeting basic human needs; 2 - Intermediate care patient (IPC): stable patient from a clinical and nursing point of view, with partial dependence on nursing professionals to meet basic human needs; 3 - High dependency care patient (PCAD): chronic patient, including palliative care, stable from a clinical point of view, but with total dependence on nursing actions to meet basic human needs; 4 - Semi-intensive care patient (PCSI): patient subject to instability of vital functions, recoverable, without imminent risk of death, requiring permanent and specialized nursing and medical assistance; 5 - Intensive care patient (PCIt): serious and recoverable patient, at imminent risk of death, subject to instability of vital functions, requiring permanent and specialized nursing and medical assistance^11^.

Given the above, this study aims to analyze the degree of dependence on nursing care by the patient classification system (SCP) in a private hospital in Porto Alegre, Rio Grande do Sul.

## Materials and Methods

This is a retrospective (historical) cohort study, with a quantitative approach, carried out in a private hospital in Porto Alegre, Rio Grande do Sul, from April 2021 to April 2022.

Rio Grande do Sul (RS) has a territorial extension of 281.730,20 km^2^, being the ninth largest Brazilian state and representing 6% of the national population. Divided into 497 municipalities, it has moderately cold winters with occasional frosts and snow and hot summers, the annual average varies between 14°C and 22°C. According to the Brazilian Institute of Geography and Statistics (IBGE), the population of RS is approximately 11.5 million, with the city of Porto Alegre being the most populous (approximately 1.5 million inhabitants)^12^^,^^13^.

**Figure 1:**
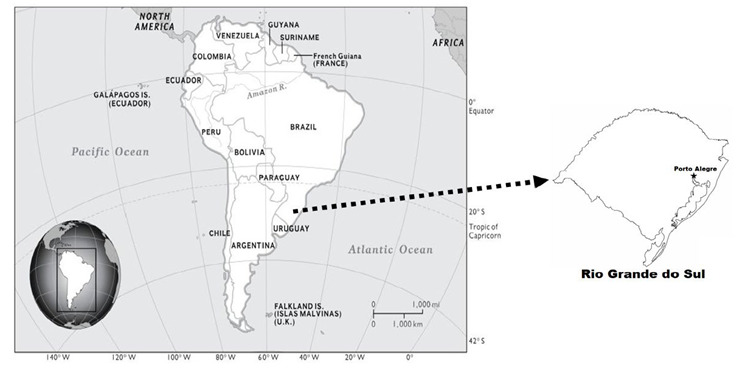
Map of South America, high lighting Rio Grande do Sul (city of Porto Alegre), Brazil

The Moinhos de Vento hospital, under study, is located in Porto Alegre (Figure 1), with 86.307 m2 of built area and 3.188 m2 of native green area, with an annual average of 16.224 hospitalizations, 16.428 surgeries, 2.497 births, and approximately the average 14 thousand daily visits. Currently, it has 30 beds in the urgent and emergency service, including reception rooms, electrocardiogram room, medication room, medical offices, red room, rooms for neurological patients, rooms for cardiological patients, rooms for patients with medium complexity, rooms for respiratory patients and isolation rooms, 72 beds in Intensive Care Units (adult, pediatric and neonatal), 304 beds for hospitalizations (clinical, surgical and obstetric), 4 rooms in the Obstetric Center, 12 rooms in the surgical center, 29 beds in the Surgical Recovery Center and 7 beds in the Obstetric Recovery Center. It is a hospital that serves patients with private plans^14^.

The research included elderly people aged 65 or over who were hospitalized due to a femur fracture and SCP was performed at the time of admission, in the middle of hospitalization, and at hospital discharge. Patients with two or more fractures, failure to perform SCP at the established times of the study, and who were admitted to the Intensive Care Center (ICU) at the time of the study evaluation were excluded.

Thus, using the non-probabilistic convenience sampling method, initially 55 patients were obtained, of which three patients had two femur fractures, seven were referred to the ICU and three had the absence of one of the assessments, thus comprising the final sample. of the research 41 patients.

Data were collected from the institution's database and electronic medical records, by study researchers from November 2022 to April 2023. For this, an instrument was used containing the following variables: sociodemographic data (sex and age), comorbidities, arrival shift (dawn 00:00 - 05:59, morning 06:00 - 11:59, afternoon 12:00 - 17:59 and night 18:00 - 23:59), boarding time (average time interval between opening of hospitalization report until arrival at the inpatient unit), risk classification, fracture characterization, location of the fracture, average time for emergency surgery, case mix, degree of dependence on nursing care upon admission, mid-hospitalization and discharge, according to the SCP, average length of hospital stay and clinical outcome (improved discharge or death).

The SCP used in the institution is a reformulation of Perroca's 15 instruments, it is divided into food, body care, dressing, walking, eliminations, mental state, skin-mucosal integrity-tissue length, motility, oxygenation, vital signs, time used to perform the dressing and therapy. For each SCP topic, there is a subdivision with a score between 1 and 4, with the higher the value the greater the degree of dependence. For better understanding, Table 1 presents the SCP topics, their subdivisions, and scores.

**Table 1:** SCP topics, subdivisions and scores

Division	Subdivision and Punctuation			
	+1	+2	+3	+4
Eating	Self-sufficient	Orally, with assistance	Through nasogastric tube	Through central catheter
Personal Care	Self-sufficient	Assistance with shower and/or oral hygiene	Shower bath, oral hygiene performed by nursing staff	Bed bath and oral hygiene performed by the nurse
Dressing	No dressing or cleaning of the wound/surgical incision, performed by the patient during bathing	Dressing performed once a day by the nursing team	Dressing performed 2 times a day by the nursing team	Dressing performed 3 times a day or more by the nursing team
Ambulation	Ambulatory	Requires assistance for ambulation	Mobility through wheelchair	Bedridden
Eliminations	Self-sufficient	Toilet use with assistance	Bedpan use	Bedpan use and bedpan evacuation, and use of a urinary catheter for diuresis control
Mental State	Oriented to time and space	Period of disorientation in time and space	Period of unconsciousness	Unconscious
Skin and Mucous Membrane Integrity - Tissue Length	Intact skin	Skin color change and/or presence of discontinuity involving the epidermis/dermis	Skin discontinuity in subcutaneous tissue and muscle. Surgical incision. Ostomies. Drains	Destruction of the dermis, epidermis, muscles, and length of other structures such as tendons and capsules
Mobility	Moves all body segments	Limited movement	Difficulty moving body segments. Passive change of position and passive movement	Unable to move any body segment. Passive change of position and passive movement
Oxygenation	Does not depend on oxygen	Continuous use of oxygen mask or cannula	Continuous use of oxygen mask or cannula	Mechanical ventilation (use of pressure or volume-controlled ventilator)
Vital Signs	Monitoring at 8-hour intervals	Monitoring at 6-hour intervals	Monitoring at 4-hour intervals	Monitoring at intervals of 2 hours or less
Time spent on wound care	No dressing or wound cleaning during bathing	Between 5 and 15 minutes	Between 15 and 30 minutes	More than 30 minutes
Therapeutics	Intramuscular or oral	Intermittent intravenous	Continuous intravenous or through nasogastric tube	Use of vasoactive drugs for blood pressure maintenance

After applying the SCP, patients are classified into: Minimum Care - 12 to 17, Intermediate Care - 18 to 22, High Dependency Care - 23 to 28, Semi Intensive Care - 29 to 34 and Intensive Care - 35 to 48. Adaptation was carried out based on the patient classification system validated by Perroca^15^.

This study respected the ethical precepts of Resolution 466/2012 and was approved by the Research Ethics Committee of the Institute of Education and Research of Hospital Moinhos de Vento, under number 3.255.989.

The data was stored in databases, made available in Zenodo^16^, and was then compiled and analyzed using SPSS® software (version 25.0, Chicago, IL Statistical Package for the Social Sciences). The type of distribution of quantitative variables was assessed using the Shapiro-Wilk test. Furthermore, the homogeneities of variances were verified using Levene's test. Arithmetic means and standard deviations were calculated for variables with normal distribution, while the median and interquartile range were calculated for those with non-normal distribution. For comparisons of quantitative variables between groups, means were compared using the repeated measures ANOVA test followed by Tukey's post-hoc test. Frequencies in percentages will be calculated for all qualitative variables. Pearson's chi-square and/or Fisher's exact tests were applied to verify possible associations between qualitative variables and the outcome studied. All estimates were two-sided with a pre-established significance level for an alpha error of 5% (p <0,05).

## Results

The sample consisted of 41 patients hospitalized with a diagnosis of femur fracture. As a result, a mean age of 84 ± 7.99 years was evidenced, with 70.73% (29) of patients belonging to the age group of 80 years or more and 29.27% (12) of patients in the age group of 65 to 80 years old, with a predominance of females, 75.61% (31).

During the period under study, the number of patients per arrival shift was obtained, with results of 4.88% (2) early morning, 29.27% (12) morning, 36.59% (15) afternoon, and 29.27% (12) night.

The risk classification in urgency and emergency indicated 32 orange patients (78.05%) and 9 yellow patients (21.95%). Fracture characterization was divided into 78.00% (32) due to fall from height, 9.80% (4) due to fall on stairs, 4.90% (2) due to wear and tear, 4.90% (2) due to fall from bed and 2.40% (1) due to movement. Regarding the location of the fracture, 53.66% (22) occurred in the femoral neck and 46.34% (19) in the trans trochanteric region.

All patients required emergency surgery, so the boarding time was 8 hours and 8 minutes, the average time from arrival at the emergency room to surgical intervention was 15 hours and 40 minutes, with 1 hour and 10 minutes as a regular time. duration of surgery, with no deaths occurring during either the emergency or hospitalization period. The average length of hospital stay was 7 days, and the measure of complexity and criticality of care, the case mix, presented an average of 2.1972 ± 0.5555 (median of 2.054, interquartile range of 1.6693 to 2.5373).

In an additional analysis, without comparing location and type of surgery, the time until surgery and average 3.87 TMPH, between 10 am and 1 pm 59 minutes: 9 and average 9.60 TMPH, between 2 pm and 5 pm 59 minutes: 7 and average 8.14 TMPH, between 6 pm and 9 pm 59 minutes: 14 and average 6.34 TMPH and between 10 pm and 25 hours 59 minutes: 4 and average 7.98 TMPH.

Regarding comorbidities, 3 deny it and 38 manifest any previous illness, namely: systemic arterial hypertension (63.41%; 26), diabetes mellitus (24.39%; 10), chronic heart failure (12.20%; 5), hypothyroidism (12.20%; 5), depression (12.20%; 5), alzheimer's (12.20%; 5), dyslipidemia (9.76%; 4), cancer (9.76%;; 4), dementia (4.88%; 2), coronary artery disease (4.88%;; 2), chronic obstructive pulmonary disease (4.88%; 2), hearing loss (4.88%; 2), arthritis rheumatoid (4.88%; 2), anxiety (4.88%; 2), stroke (4.88%; 2), Parkinson's (2.44%; 1), gastroesophageal reflux disease (2.44%; 1), amyotrophic lateral sclerosis (2.44%; 1), chronic kidney disease (2.44%; 1), arrhythmia (2.44%; 1), subarachnoid hemorrhage (2.44%; 1), secondary obstructive hydrocephalus (2.44%; 1), osteoporosis (2.44%; 1), hepatic encephalopathy (2.44%; 1), ischemic myocardia (2.44%; 1), seizures (2.44%; 1), obesity (2.44%; 1), anxiety and percutaneous transluminal coronary angioplasty (2.44%; 1), ischemic heart disease (2.44%; 1), pulmonary thromboembolism (2.44%; 1) and atrial septal defect (2.44%; 1).

Table 2 shows the total median length of stay with the number of patients according to the number of comorbidities, in addition to a more in-depth analysis of the three main comorbidities observed, namely: Systemic Arterial Hypertension (SAH), Diabetes Mellitus (DM), and Congestive Heart Failure (CHF).

**Table 2:** Number of Comorbidities by Relationship of Number of Patients and Average Length of Hospitalization

Amount of Comorbidity	Absolute Frequency and Permanence			
	N° of Patients	1st Quartile	Median	3rd Quartile
0	2	4.20	4.50	4.70
1 to 2	24	4.00	6.00	7.20
3 to 4	11	4.00	6.00	8.00
5 or more	4	5.20	13.00	20.50
With Only HAS, among the main	20	6.00	6.00	9.50
With Just DM, among the main	1	9.00	9.00	9.00
With Only HAS and ICC, among the main	5	4.00	4.00	4.00
With Only HAS and DM, among the main	5	4.00	4.00	4.00
No presentation of any of the main	14	4.00	5.00	8.00

SAH: Systemic Arterial Hypertension; DM: Diabetes Mellitus; CHF: Congestive Heart Failure.

Thus, the degree of dependence on nursing care, according to the SCP, showed an average of 24.46 upon patient admission, ranging from 14 (minimum care) to 29 (semi-intensive care), an average of 26.12 in the intermediate assessment, ranging from 18 (intermediate care) to 33 (semi-intensive care) and an average of 26.24 during the patient's discharge period, with a range from 21 (intermediate care) to 33 (semi-intensive care). Table 3 shows the average for each subdivision of the SCP, carried out during the three periods under study. There was a significant increase in the indicators of ambulation and skin integrity-mucosa-tissue length when comparing the three assessments.

**Table 3:** Mean and standard deviation of the score for each indicator of the Patient Classification System, according to the moment of assessment

Indicators	Punctuation First Assessment	Punctuation First Assessment	Punctuation Last Assessment	p-value*
	Mean ± SD	Mean ± SD	Mean ± SD	
Eating	1.48 ± 0.54a	1.51 ± 0.54a	1.51 ± 0.54a	0.51
Personal Care	3.46 ± 0.91a	3.41 ± 0.88a	3.41 ± 0.82a	0.47
Dressing	1.21 ± 0.41a	1.58 ± 0.49a	1.63 ±.0.48a	0.15
Ambulation	1.17 ± 0.43a	3.48 ± 0.82b	3.39 ± 0.82b	0.01
Eliminations	3.34 ± 0.84a	3.19 ± 0.83a	3.21 ± 0.64a	0.41
Mental State	1.39 ± 0.61a	1.41 ± 0.53a	1.39 ± 0.53a	0.53
Skin and Mucous Membrane Integrity - Tissue Length	1.29 ± 0,45a	2.00 ± 0.91b	2.14 ± 0.89b	0.04
Mobility	2.36 ± 1.00a	2.26 ± 0.69a	2.29 ± 0.67a	0.65
Oxygenation	1.09 ± 0.36a	1.17 ± 0.48a	1.14 ± 0.41a	0.23
Vital Signs	2.02 ± 0.46a	2.21 ± 0.71a	2.14 ± 0.56a	0.25
Time spent on wound care	1.24 ± 0.57a	1.70 ± 0.70a	1.80 ± 0.70a	0.08
Therapeutics	2.00 ± 0.31a	2.14 ± 0.47a	2.12 ± 0.39a	0.33

*SD: Standard deviation *The ANOVA test for repeated measures was applied followed by Tukey's post-hoc test.*

Letters (a,b): repeated letters indicate that there are no statistically significant differences (p >0.05), while different letters indicate statistically significant differences (p <0.05).

Table 4 also shows the classification of care using the SCP at each moment stipulated by the study, without comparison with the time each patient was hospitalized.

**Table 4:** Care classification categories, according to the patient evaluation period patient

Patient Classification System	First Assessment	Assessment Intermediate	Last Assessment	p-value*
	n = 41	n = 41	n = 41	
Minimum Care	2 (4.88)	-	-	0.88
Intermediate Care	3 (7.32)	3 (7.32)	2 (4.88)	0.75
High Dependency Care	33 (80.49)	26 (63.41)	28 (68.29)	0.36
Semi-Intensive Care	3 (7.32)	12 (29.27)	11 (26.83)	0.38
Intensive Care	-	-	-	

*Pearson's chi-square and Fisher's exact tests were applied as appropriate.

Table 5 analyzed the number of patients, classified by sex and age group, in each evaluation carried out, without considering the length of stay. In the last evaluation of each patient, the length of stay and clinical outcome were also highlighted. This comparison regarding the total number of each hospitalization created the possibility of observing that there was the greatest increase in the classification of dependence on care in females and patients over 80 years of age showed an increase in their level of care.

**Table 5:** Distribution of the change in the absolute number of patients by sex, age group during each evaluation, and length of stay with clinical outcome in the last evaluation of each patient

Variables	Data According to Each Assessment				
	Minimum Care	Intermediate Care	High Dependency Care	Semi- Intensive Care	p-value*
1st Assessment					
Gender					0.27
Masculine	2 (4.88)	1 (2.44)	6 (14.63)	1 (2.44)	
Feminine	-	2 (4.88)	27 (65.85)	2 (4.88)	
Age Range					0.30
65 to 80 Years	2 (4.88)	-	10 (24.39)	-	
80 years or more	-	3 (7.32%)	23 (56.10)	3 (7.32)	
2nd Assessment					
Gender					0.28
Masculine	-	2 (4.88)	6 (14.63)	2 (4.88)	
Feminine	-	1 (2.44)	20 (48.78)	10 (24.39)	
Age Range					0.90
65 to 80 Years	-	1 (2.44)	8 (19.51)	3 (7.32)	
80 years or more	-	2 (4.88)	18 (43.90)	9 (21.95)	
3rd Assessment					
Gender					0.62
Masculine	-	1 (2.44)	7 (17.07)	2 (4.88)	
Feminine	-	1 (2.44)	21 (51.22)	9 (21.95)	
Age Range					0.54
65 to 80 Years	-	-	10 (24.39)	2 (4.88)	
80 years or more	-	2 (4.88)	18 (43.90)	9 (21.95)	
Length of stay					0.80
Up to 5 days	-	1 (2.44)	18 (43.90)	5 (12.20)	
6 to 10 days	-	1 (2.44)	7 (17.07)	3 (7.32)	
11 to 20 days	-	-	3 (7.32)	2 (4.88)	
20 days or more	-	-	-	1	
Outcome					0.24
Hospital Discharge	-	2 (4.88)	28 (68.29)	11 (26.83)	
Death	-	-	-	-	

*Pearson's chi-square and Fisher's exact tests were applied as appropriate.

## Discussion

About the sociodemographic profile, in this study, a predominance of females was observed. The finding corroborates the results of the study carried out at the teaching hospital in the central region of São Paulo^17^, which showed that 75.22% of the 113 patients treated were women. Another characteristic of the patient profile is age, the descriptive and comparative study^18^ recorded a mean age of 70.94 ± 7.50 years for patients who were at high risk of falling during their hospitalization. Therefore, it is extremely important to study age, with the average age being 79 years among patients at the teaching hospital^17^, considering elderly people over 60. In the present analysis, the average was 84 years old. This difference is probably because this study considered elderly patients over 65 years of age.

The reasons for falls are varied, being intrinsic and extrinsic factors. The reasons for falls are varied, being intrinsic and extrinsic factors. A study evaluating 104 patients indicated that 77.60% suffered a fall from the same level and 31.30% from a different level^19^. The results strengthen the findings of the present study, which recorded that 78% of falls are from standing height and 9.80% fall from stairs.

In this sense, the location of the fracture occurs, occurring 53.66% in the femoral neck and 46.34% in the trans trochanteric region. The result is in line with what was demonstrated in the study mentioned above^19^, with the greatest evidence of femoral neck fracture at 35.60% (37), followed by trans trochanteric fracture at 19.20% (20).

Patients treated surgically at Hospital São Paulo - Unifesp^20^ presented an average of 5.80 days for the time from fracture to surgical intervention. Of the 269 patients, only 13.01% underwent surgery within the first 24 hours. This study differs from current research, as only one patient underwent surgery after 24 hours, with the overall average surgery time for the 41 patients being 15 hours and 40 minutes. It should be considered that the hospital under study is a private hospital and Hospital São Paulo - Unifesp primarily serves patients from the Unified Health System. There was no association in this study between time for surgery and length of stay, as there are several factors that influence the results.

Associated with advanced age are patients' underlying comorbidities. A study carried out with 233 hospitalized elderly people^21^, aged 65 years or over, found the presence of hypertension in 45.9% (107) and DM in 35.2% (32) of patients, in addition to highlighting that in the total survey, 87.5% of individuals had at least one comorbidity. In our study, hypertension was manifested in 58.54% (24) and DM in 21.95% (9), successively. It was also shown that only 3 patients did not have any comorbidity, thus, 92.68% (38) had 1 or more comorbidity, highlighting that 50% of patients had 3 or 4 comorbidities.

Of the indicators analyzed in this study, about the degree of dependence on nursing care, according to the patient classification system, the following were highlighted as the highest: body care with 70.73% in the 1st assessment, 65.85% in the 2nd and 63.41% in the 3rd assessment, such as bed bath and oral hygiene performed by the nurse (4 points). Eliminations with 39.02% in the 1st assessment, 48.78% in the 2nd, and 60.98% in the 3rd, such as the use of a bedpan or eliminations in bed (3 points). Motility with 51.22% in the 1st and 2nd assessment and 53.66% in the 3rd assessment, as movement limitation (2 points). Ambulation, as restricted to bed (4 points) 0.00% in the 1st assessment, 70.73% in the 2nd, and 60.98% in the 3rd. Like the minors, oxygenation counts at 92.68% in the 1st assessment and 87.80% in the 2nd and 3rd non-oxygen dependent patients (1 point). Food 53.66% in the 1st assessment, 51.22% in the 2nd and 3rd as self-sufficient (1 point). Dressing 21.95% in the 1st assessment, 58.54% in the 2nd, and 63.41 in the 3rd as dressing performed once a day by the nursing team (2 points). Mental state 65.85 in the 1st assessment, 60.98% in the 2nd, and 63.41% in the 3rd orientation in time and space (1 point).

These data were compared with the research entitled “Grau de dependência de idosos hospitalizados conforme o sistema de classificação de pacientes”^22^, in the Geriatric Inpatient Unit of the São Lucas Hospital of the Pontifical Catholic University of Rio Grande do Sul (PUCRS). It assessed the degree of dependence of patients with a classification partially similar to the indicators at Hospital Moinhos de Vento analyzed in the current research, however, it was not carried out in patients with femoral fractures.

In the first assessment, the indicators with the highest scores were body care, elimination, and locomotion. In our study, the first assessment highlighted body care, eliminations, and motility with the highest scores, with oxygenation, ambulation, and dressing the lowest. In study^22^ carried out at PUCRS, the highest rates match our results, however, like the lowest rates we have a difference in vital signs and behavior.

In the second assessment, walking, body care, and elimination led to the highest rates, with oxygenation, mental state, and nutrition being the last. In the study under discussion, the highest indices are confirmed, differing only in two from the lowest ones, namely, vital signs and behavior. Finally, in the last evaluation under study, body care, ambulation, and elimination remained higher, and oxygenation, mental state, and nutrition remained higher in the smaller ones. The older ones confirm the results; however, the smaller ones differ in two aspects: vital signs and behavior. The results obtained between the articles under discussion present significant differences when comparing the smallest indicators because they use, as previously stated, partially different indicators.

With the complete indicators, the total average is calculated to divide the care category, namely: minimum care, intermediate care, high dependency care, semi-intensive care, and intensive care. The results of the study showed that high dependency care (23 to 28 points) remained in greater quantity during the three assessments, mainly because they presented high rates of assistance in ambulation, body care, and eliminations. There was a lack of minimum care during intermediate and final assessments, as well as a lack of intensive care throughout the hospital nursing process, since elderly people with femoral fractures show rates above 1.17 in the 12 indicators, leading to passing the classification above the minimum level of care (12 to 17 points). The increasing number of intensive care between the first assessment and the last one stands out, with its peak in the middle, because the patient is more weakened and has an understanding of their physical state. This data is similar to that found in research published in the Revista Brasileira de Enfermagem (REBEn)^22^ which found a greater presence of intermediate care during the three assessments and did not present intensive care during hospital nursing care.

The prolonged period of hospitalization has a negative impact on the elderly, contributing to the emergence of health problems in the hospital environment, such as infectious processes, pressure injuries, and increased mortality rates^23^. The epidemiological, retrospective, and quantitative study^24^, carried out using secondary data obtained from the Hospital Information System of the Unified Health System (SIH/SUS), made available by the SUS Information Technology Department (DATASUS), which recorded 291.369 hospitalizations between 2016 and 202 0 in Brazil, saw the average length of stay at around 8.5 days, with 7.5 being seen in the South of Brazil. This data is similar to that found in this research, in which the average length of hospital stay was 7 days, varying from 2 to 22 days. In addition, in the last evaluation carried out in the study, a greater number of patients were found in high-dependency care, in which, in abundance, they spent 0 to 5 days in the hospital.

In the analysis, the clinical outcomes were satisfied, with no deaths during the hospitalization period. This finding differs from those found, such as the study at the Army Central Hospital^25^, in which there were 16.7% (33) deaths. Also noteworthy is the Brazilian study^24^, in which it verified hospitalizations over 4 years, that of 291.369 patients, 15209 (5.22%) died.

Therefore, non-probabilistic convenience sampling is considered a limitation of this research (55), which allowed for a descriptive study, but made it impossible to make inferential statistical comparisons, due to the low statistical power of the sample. Another limitation was that the study location was in a private hospital, and therefore the findings must be taken into consideration with caution, as they may not represent the reality of public hospitals.

## Conclusion

Elderly people hospitalized for femoral fractures require, for the most part, highly dependent care, according to the average, ranging from a score of 23 to 28, that is, they lack care about food, body care, dressings, walking, eliminations, mental status, skin-mucosal integrity-tissue length, motility, oxygenation, vital signs, time used to perform the dressing and therapy.

These data demonstrated results, according to the adaptation of Perroca's original instrument, for classifying the needs of elderly patients with femoral fractures. The study provides specific knowledge to carry out the care of the in-hospital nursing process, thus allowing the systematization of the nursing team's assistance, which can be used in management practice, carrying out the appropriate classification and providing the correct workload for elderly people with fractures of the femur.
